# “They have been neglected for a long time”: a qualitative study on the role and recognition of rural health motivators in the Shiselweni region, Eswatini

**DOI:** 10.1186/s12960-020-00504-9

**Published:** 2020-09-21

**Authors:** Caroline Walker, Doris Burtscher, John Myeni, Bernhard Kerschberger, Bernadette Schausberger, Barbara Rusch, Nosipho Dlamini, Katherine Whitehouse

**Affiliations:** 1 Médecins Sans Frontières, Mbabane, Eswatini; 2 Vienna Evaluation Unit/Anthropology, Médecins Sans Frontières, Vienna, Austria; 3 Prevention and Promotion Programme, Ministry of Health, Mbabane, Eswatini; 4 Médecins Sans Frontières, Geneva, Switzerland; 5 Luxembourg Operational Research Unit (LuxOr), Médecins Sans Frontières, Brussels, Belgium

**Keywords:** Community health worker, CHW, Rural health, Health promotion, HIV, Gender, Southern Africa, Public health, Primary healthcare, Swaziland

## Abstract

**Background:**

Community health workers (CHWs) are increasingly engaged to address human resource shortages and fill primary healthcare gaps. In Eswatini, a cadre of CHWs called Rural Health Motivators (RHM) was introduced in 1976 to respond to key public health challenges. However, the emergence of health needs, particularly HIV/TB, has been met with inadequate programme amendments, and the role of RHMs has become marginalised following the addition of other CHWs supported by non-governmental organisations. This study was implemented to understand the role of RHMs in decentralised HIV/TB activities. In this paper, we explore the findings in relation to the recognition of RHMs and the programme.

**Methods:**

This exploratory qualitative study utilised individual in-depth interviews, group and focus group discussions, participatory methods (utilising a game format) and observations. Participants were purposively selected and comprised RHM programme implementers, community stakeholders and local and non-governmental personnel. Data collection took place between August and September 2019. Interviews were conducted in English or siSwati and transcribed. SiSwati interviews were translated directly into English. All interviews were audio-recorded, manually coded and thematically analysed. Data was validated through methodical triangulation.

**Results:**

Suboptimal organisational structure and support, primarily insufficient training and supervision for activities were factors identified through interviews and observation activities. Significant confusion of the RHM role was observed, with community expectations beyond formally endorsed tasks. Community participants expressed dissatisfaction with receiving health information only, preferring physical assistance in the form of goods. Additionally, gender emerged as a significant influencing factor on the acceptability of health messages and the engagement of RHMs with community members. Expectations and structurally limiting factors shape the extent to which RHMs are recognised as integral to the health system, at all social and organisational levels.

**Conclusions:**

Findings highlight the lack of recognition of RHMs and the programme at both community and national levels. This, along with historical neglect, has hindered the capacity of RHMs to successfully contribute to positive health outcomes for rural communities. Renewed attention and support mechanisms for this cadre are needed. Clarification of the RHM role in line with current health challenges and clearer role parameters is essential.

## Introduction

Shortages of qualified health professionals are a significant challenge to efficient and effective health system functioning [1–9]. This is especially pronounced in lower/middle-income countries and hard-to-reach areas [1, 2, 10–12]. Community health workers (CHWs) emerged as a solution to extend the provision of social services and focus on the “promotion of preventive or curative primary health services” [3, 4]. In Sub-Saharan Africa, CHWs have been engaged to address human resource shortages to improve access to basic healthcare [1–8], including HIV services [13]. This has led to increasing recognition of the integral role CHWs have in health systems [1, 3–5, 7, 14, 15]. Yet, globally, difficulties persist for this cadre including fragmented services [15], poor integration with the health system [15–17], insufficient support [18], low remuneration [13, 19] and lack of training [15, 19].

Eswatini faces a myriad of health challenges [4, 8, 20]. In 1976, to respond to the country’s main primary health needs (e.g. hygiene, nutrition, infant mortality), the Ministry of Health (MoH) established a national CHW programme called Rural Health Motivators (RHM). The main characteristics of the programme are covered in Table 1. More recently, the programme has focused on health promotion messages, household assessments and general community health referrals [21]. With the declaration of a national HIV emergency in 1999 [22], the RHM programme incorporated HIV- and tuberculosis (TB)-related responsibilities [22, 23].

**Table 1:** Main characteristics of RHM programme

• Over 5 000 RHMs in the country (majority female)	
• The programme aims to reach every household in the country	
• RHMs are chosen by community leaders and work in their home communities	
• MoH gives a monthly stipend of 350 Swazi Emalangeni (approximately US$ 24)	
• The programme stipulates working hours of 2.5 days per week	
• Main RHM responsibilities:	
o Visiting homesteads/households	
o Referrals to health facilities	
o Health education on hygiene, nutrition, antenatal care, immunisation and HIV/TB	
o Community-based growth monitoring	
o First aid	

Certain elements of the programme have been highlighted for further development. RHM leads were introduced to improve supervision [15] and given reporting responsibilities in addition to regular tasks [12], yet elaboration on support mechanisms is still needed [3, 4]. The existence of parallel donor-funded CHWs [4] performing HIV/TB-specific tasks has resulted in ambiguity around the RHM role. Low remuneration [4] highlights tensions in the role of volunteer versus compensated CHW [15]. Lastly, community trust of the group could be improved, partly influenced by the predominantly female RHM workforce [3], RHMs working in home communities [3] and training [4].

The study aimed to understand the role of RHMs in decentralised HIV/TB activities. Due to the above shortfalls, we will focus on the RHM experience of programme recognition and the understanding of their role by key stakeholders, including community members, local leadership and government structures. This study intends to inform the MoH on future iterations of the RHM programme and health policy, assist NGOs on how best to support RHMs and inform CHW programmes in comparable settings.

## Methods

### Study design

This study used a qualitative research design with an exploratory approach [24]. Data was gathered between August and September 2019, using individual in-depth interviews (IDIs), participant observation, focus group discussions (FGDs) and group discussions including participatory methods to understand the different perspectives of participants.

### Setting

Criteria for study site selection included two health zones of Shiselweni Region, to allow for comparison and contrast of RHM experience between remote areas in each zone. Remote areas in these zones were chosen at random. Both areas are a substantial distance from the main roads and have poor transport availability. Disparity of access to health services distinguished the areas; one had a primary healthcare clinic while the other had a significant distance to any health services.

The predominantly rural Shiselweni Region has approximately 210 000 inhabitants with an estimated HIV prevalence of 25.9%—36 000 people living with HIV [25]. Shiselweni comprises three health zones with each served by one secondary care facility and approximately eight primary care clinics. An estimated 1 200 RHMs operate in the region under the supervision of three RHM trainers.

### Study population

To explore the research question from a variety of angles, the study population comprised a number of key groups of participants: RHMs and RHM trainers; community leaders, community members and people living with HIV; Médecins Sans Frontières (MSF) staff; MoH nurses and staff from MoH; and Ministry of Tinkhundla Administration and Development (MTAD).^1^ The study population characteristics are detailed in Table 2.

**Table 2:** Characteristics of the study participants per methodology

Methodology of data collection	Participants	Number of interviews
IDI	RHM	8
	MoH nurse	2
	Community leader	2
	RHM trainer	2
	MSF staff	2
	MoH (key informant)	2
	MTAD (key informant)	1
		*Total of 19 participants*
FGD	Women	2
	Men	1
	People living with HIV (women)	1
		*Total of 29 participants*
Group discussions (using participatory methods)	RHMs	1*
		*Total of 5 participants*
Groups discussion	Men	1
		*Total of 3 participants*
Observation	RHMs	4*

*Observation and participatory methods were done with RHMs already interviewed, in order to further develop a trusting relationship and to add depth to the understanding of IDI discussions

### Sampling and recruitment

Purposive sampling was used to identify eligible research participants with stakeholder sampling based on those most involved in the RHM programme [26]. Prior to recruitment, RHM trainers and community leaders were approached to explain the study and to enlist their support in mobilisation for participation. They were also engaged as participants in the study due to their leadership roles in the chosen areas. Those identified were invited to participate in IDIs organised in relevant community settings by research assistants and MSF staff working in Shiselweni communities. The criteria for RHM participation were those actively working in the selected areas and willing to participate. Efforts were made to have participants reflective of the RHM workforce—covering a variety of ages giving a range in years of experience. No male RHMs worked in the selected areas; thus, only female participants were engaged. Following an IDI, RHMs were further invited to participate in observation and group discussions. IDIs with MoH and MTAD participants were arranged by the RHM programme manager based on their positions in the relevant departments and knowledge of community activity and the country’s health system. IDIs with MoH nurses working at the closest health facility to each area were organised by MSF staff, while IDIs with MSF staff were organised by PI based on their experience working with RHMs.

### Data collection

IDIs were used with all participants to gain a broad perspective of the RHM role and to understand the social and organisational context in which they work. FGDs were used to gather views of the community (both men and women) and people living with HIV, to allow an in-depth understanding of RHM work and community reception. Observation was used to gain a stronger understanding of RHM activity and community engagement. As understanding the RHM experience was paramount, a group discussion using participatory methods was conducted with RHMs [27]. Mixed data collection methods were applied for RHMs for a better understanding of their work, challenges and recommendations.

Interviews were conducted in English and siSwati, depending on the participants’ preference. Two female research assistants, fluent in siSwati and trained in qualitative research, conducted the siSwati interviews. Interview guides for IDIs, FGDs and group discussions were developed and discussed in-depth with the research team, and additional themes were added if needed from interview experience. After each siSwati interview, the principal investigator (PI) debriefed with research assistants to discuss the main themes, translation issues or difficult questions.

An observation template was used in order to incorporate additional aspects of the RHM perspective. The participatory method discussion was conducted in a comfortable and open environment. A game format was utilised, designed by the PI and pre-tested with the MSF community team.

### Data analysis

All interviews were transcribed verbatim by the research assistants (interviews in siSwati translated directly into English) and PI (interviews in English). Notes from observations, participatory exercises and additional notes taken during the research were typed. Research assistants validated each other’s siSwati interview transcripts, and the overall quality control and assurance of all transcripts were done by a co-investigator experienced in qualitative research and the context.

Data was coded manually by the PI, developing codes, categories and themes from the data. This approach allowed for comparisons and contrasts to be made amongst transcripts [28, 29] and for key issues to be identified [29]. To ensure consistency of the data analysis, continuous discussions were held between the PI and co-investigators to explore emerging themes and develop coding schemes. For supporting analysis, the PI discussed findings with members of the MSF community team.

Data validation was ensured through methodological triangulation [24] and continuous reflection and discussion within data validation workshops. Workshops were held with RHMs to assess the extent to which research findings were representative of their perspectives. Contributions from these discussions were included in the analysis and recommendations, highlighting the areas for improvement.

### Ethics and informed consent

The study protocol was approved by the MSF Ethics Review Board and Eswatini’s National Health Research Review Board. Informed consent forms were available in English and siSwati, with all participants providing written informed consent before study enrolment. The consent process was also explained in siSwati when relevant.

## Results

This study identified misunderstandings of the RHM role and the structural limitations that reduce their effectiveness. These two components underpin and influence the extent to which the programme is recognised and accepted at the health system, community and individual levels. Gender further influences how the community experiences and recognises the work of RHMs.

### Understanding of the role

#### The RHM role: “their visibility is not clear”

Confusion over the exact role of RHMs was widespread amongst all study participants, including MoH representatives. Some common understandings of the focus on sanitation and hygiene emerged, yet numerous additional responsibilities were identified ranging from looking after sick people to “giving pills”. The historical development of the RHM programme was noted as a reason for this confusion at the community level, with many comparisons made between the original and current role. Regarding HIV/TB work, little was mentioned without probing. Historical inclusion of home-based care for HIV was occasionally stated, with challenges on disclosure and promoting condom use often cited. Older community members were more likely to know RHMs personally. Observation showed RHMs focus on older community members in household visits, with the exception of babies.

### Expectations: “when you are going to check on a sick person, bring a hoe”

There is an expectation for RHMs to work past their MoH-endorsed role. Additional tasks included the distribution of food parcels, collection of money and washing dead bodies. Such tasks affect RHM relationships with community members, the latter perceiving RHM bias in choosing “favourite” households for the distribution of food. Expectations were identified at the community, clinic and governmental levels, with some RHMs being asked to clean clinics.

Community members expressed an understanding and expectation for RHMs to provide more than health education and clinic referrals:

When my wife was sick, [the RHM] used to come to my home, even when she was weeding she could come and assist her, the role of the RHM stayed in my heart as a big thing. (Male community member)

Various reasons for poor recognition emerged, including that RHMs no longer provide first aid material (historically distributed). One RHM emphasised that community members complain:

A person will come when you are teaching and tell you they have a headache, you do not have paracetamol, so when you are teaching, they will not listen because there is nothing that you have helped them with. (Female RHM)

During IDIs, RHMs were positive about the reception from community members, only discussing these challenges more openly in group discussions. Community expectations and confusion over what health education entails lead to assumptions of RHMs doing work for community members, e.g. building a latrine after education on sanitation and hygiene. One RHM explained this, including the influence community leaders have on community expectations:

The way [community leaders] say it, it sounds like we are supposed to do it for the people. The people will also think that a RHM is someone that has nothing to do. (Female RHM)

### Organisational structure of the RHM programme

Several aspects related to the organisation of the RHM programme emerged from the data. It was identified that RHMs could absorb new activities and deliver health programmes more effectively with appropriate training and materials provided. The MoH and NGOs do not consistently provide RHMs with sufficient training, materials and support needed to perform required tasks.

Comparing RHMs with Active Case Finders (ACF) of the TB programme, a key informant (KI) explained the success:

Ask anything to an ACF right now they give you… the knowledge the ACF has it is more than what the RHM has. Because we trained them. (KI, MoH)

Following the initial training, RHMs reported that they felt neglected. Accessing formal programme support structures was a common challenge. In particular, a lack of supervision was mentioned, with several RHMs explaining that they needed more supervision to have their work validated or corrected.

The large number of RHMs and the ratio to supervisors were highlighted by MoH KIs as a limitation in the programme structure.

… For the RHM programme manager to manage 5,000 people, that’s a very big group of people. (KI, MoH)

The RHM programme falls under the MoH. A MoH KI admitted to not knowing what the RHM job currently is.

I’m not sure, honestly I just have to be honest, I’m not sure (KI, MoH)

Participants reported a lack of collaboration between national and regional levels, with current MoH structures hindering RHM performance and programming.

They are so many that their data should have an impact in the country. But from what I’ve seen, it seems the national level has to come up with a system… (KI, MoH)

The lack of organisational structure was also seen in the absence of clarity on HIV/TB activities. KIs and MSF staff explain how other CHW groups like the ACFs were given TB-specific tasks and relevant training, leaving the RHM role unclear in terms of HIV/TB. The position of the RHM programme within the broader MoH system was clearer at the peak of the HIV crisis, compared to the current low prioritisation of the programme:

We were working together but it was then, when HIV was a problem, we had good relationship because we wanted to see them taking care of the sick… (KI, MoH)

Insufficient stipends, termed “soap money”, were commonly cited as a challenge and often affected the RHMs’ ability to present themselves as professionals in the community. The irony in terming it a “salary” was acknowledged. MoH informants discussed how this could affect motivation, with the need to cover transport costs leaving some at a loss each month. RHMs considered insufficient stipends as a lack of “consideration” for them and their work:

We are wondering if the [government] may consider us and give us a bit of something so that even as I go along the mountains, it may feel easy to walk if there is money making me happy (RHM)

Unfortunately my sneakers are worn out. You wouldn’t want to arrive to people dressed like that, you need to dress better (Female RHM)

### Recognition

#### Self-recognition: “It’s something in the heart, you just get happy that you have done the visit” (RHM)

Many RHMs portrayed their work as significant, explaining it as an “act of service”. Numerous RHMs invoked religious language when describing their work, often involving elements of benevolence. Responding to socio-economic challenges, the majority of RHMs discussed sharing their own food with community members under their care and paying for transport to clinics.

Contrasting with this, however, RHMs saw limitations in their efficacy. For HIV, they emphasised that “nothing” can be done but to motivate people to go to the clinic where doctors could provide substantial help.

The stories [health promotion], they already know, we have already told them, only we have nothing helpful … (Female RHM)

#### Visibility and respect for RHMs: “we have them, but we do not see them”

There is consensus that no “one” type of RHM exists, with commitment to work schedule, skill and knowledge varying amongst the group. For RHMs to be successful, community members felt RHMs needed to work every day. On average, RHMs are tasked with 15–20 households to visit each month. However, some households were never visited by their RHM.

Support from community leadership was seen as vital for the success and ease of RHM work. Many RHMs felt unsupported by community leaders, with some leaders seeing RHMs as “earning for free”. There are not always opportunities to conduct health education at community meetings. A community leader said he was unable to remark on how well RHMs are working and that he did not have their phone numbers. Challenges including the old age of RHMs and low education levels, especially regarding updated MoH criteria, were common.

These days we are living in times where people are educated, it seems even the RHMs could be people who are at a level of the people … Today I feel like the RHMs should be upgraded, able to look at things that are written down. (Community leader)

### The experience and influence of gender

Gender emerged as a fundamental component in both the content of RHM health education and the acceptability of interacting with the group. Poor male health-seeking behaviour compared to women was often identified as problematic. The programme was regularly perceived by community members as dealing with “woman issues.”

Engaging men was explained as a big challenge, with several examples of men rarely prioritising interactions with RHMs, as they are too busy with other tasks—“men do not like to sit and listen”. Instead, they instruct their wives to meet with RHMs. Gender was seen as potentially influencing the ease and acceptability of discussing certain topics, both for RHMs sensitising community members on specific health issues and for members of the community to raise their individual health needs with RHMs. Community leaders echoed this widespread opinion, explaining that female RHMs are appropriate to speak to women and that men would accept information better from male RHMs.

…obviously the man will think that the female RHM could go and tell his wife that your husband has had this, whereas if it is a man he could easily understand. (Community leader)

It is difficult to take my issue and share it with somebody’s wife [RHM]. (Male community member)

In contrast, higher-level MoH participants did not consider gender as a limiting factor on the acceptability of health messaging in the community.

I do not think there is difficulty in talking to each other for whichever gender when it comes to seeking health related information. (MoH nurse)

I think any gender of RHMs can work. (KI, MoH)

In addition to gender influencing whether RHMs can discuss certain topics, one RHM highlighted the difficulty of changing behaviour in topics related to gender (e.g. condom use) by comparing to her personal life.

With HIV, I think not using condoms [is the biggest problem] … I usually talk about that to people as an RHM, but I am failing to do that inside my own house. Even with my own husband, it is difficult for us [women] to use a condom … (Female RHM)

## Discussion

Overall recognition of RHMs appeared as the main emerging theme in this study exploring the role of RHMs in Eswatini. We identified that the lack of structural recognition and understanding of RHM realities influences RHM work, hindering positive health outcomes of communities. In relation to recognition, we discussed the ambiguity of the role, gendered dynamics in community interactions and organisational structure and support.

### Ambiguity of the role

We identified several ways in which RHMs occupy an ambiguous position in the Eswatini health system, with confusion around the role recorded amongst all study participants. Since the introduction of the RHM programme, the country’s health landscape has changed significantly, inextricably linked to the emergence of HIV/TB. The lack of RHM recognition is exemplified specifically by their indistinct role on HIV/TB. Responding to priority health issues was the original aim of the RHM programme at its inception. However, the introduction of other donor-funded CHW groups, e.g. the ACFs focused on TB, illustrates the marginalisation of RHMs on pertinent public health issues.

Although MoH informants detailed the role of RHMs as prevention and health information-focused, this study identified considerable scepticism on their effectiveness. Previous distribution of items, including first aid, as well as the willingness of RHMs to perform tasks outside their remit, may have created unrealistic community expectations and confusion over the position.^2^ RHMs emphasised this during data validation workshop discussions. A previous study on volunteer health workers in Eswatini highlighted the detrimental impact on work that a lack of role clarity can have [30]. This supports our findings that clearer parameters of work are needed for effective implementation of community health interventions:

The hasty nature in which expert clients were engaged, in the midst of an AIDS emergency, resulted in a lack of consensus on the scope of work and definition of the position of expert clients within the government structure [30]

Our findings have implications for the administrative structures to clearly delineate roles and responsibilities in light of health needs such as HIV/TB. This needs to be communicated across government and community levels, including amongst RHMs.

### Gender dynamics and community interactions

This study clearly identified the social and cultural influence of gender on how the RHM programme is viewed by community members, as well as the ease for RHMs to fulfil their tasks. Globally, insufficient attention has been paid to gender in CHW programmes [3, 31–33], and in this context, gender has been recognised as a specific challenge [34, 35]. The introduction of male RHMs has been recommended to increase the reach and acceptance of health messaging for male community members [3, 5]. The impact of gender in how community members view health and its impact on health choices (e.g. the RHM having difficulty to negotiate condom use with her husband) cannot be understated. Gender needs to be considered when designing public health interventions, e.g. the presence of more men in the programme might address widespread notions that community health work is for women and deals with “women’s issues” only. However, careful attention must be paid to not further compound gender inequality [36, 37].

### Organisational structure and support

Insufficient recognition of the RHM role in public health structures was illustrated in this study through numerous examples, including low remuneration, limited opportunities for regular quality supervision, suboptimal training and other forms of formal support such as uniforms. This was also repeated in data validation workshops, with requests made for this to be addressed. The low stipend and suboptimal structural support may be understood at an institutional level as a symbolic lack of RHM recognition. RHMs reported that in addition to a sufficient stipend, they needed their work to be *considered*—for higher levels to understand their work and challenges and provide relevant support*.* This is further understood as RHMs not being consulted in programme design or programme design not reflecting RHM realities. Discussions during data validation workshops emphasised the neglect many RHMs felt, related to both supervision and unfeasible work schedules. Studies have identified the impact of poor structural support: links between insufficient compensation and negative outcomes, including psychological distress [38] or financial compensation as a key motivator for CHW performance [39, 40]. However, monetary recognition is not consistently seen as a singular motivator for work. Supportive supervision has shown to address challenges of the recognition and improve work output [1, 5, 40, 41], and regular training is positively associated with CHW confidence in their role [1, 42]. In order to enhance institutional recognition of the programme, and make further positive health contributions at the community level, increased structural support is needed, designed in consultation with RHMs.

## Recommendations

Globally, the lack of recognition of CHWs in MoH programmes has been cited as a challenge [19, 40, 43–45]. Increased recognition is seen as potentially improving lay health worker performance [41]. In this context, the lack of recognition and historical neglect have hindered the capacity of RHMs to successfully contribute to positive health outcomes for rural communities. Improved recognition might be achieved through the clarification of the RHM role in alignment with current public health challenges. In light of the country’s cultural landscape, increasing the number of male RHMs may contribute to improved uptake of refined health messaging [3]. Adjusted ratios between community-based and supervisory positions, with strengthened supervision and stipends, would possibly address inefficiencies of current structural support mechanisms and improve the overall efficiency of this key group. Community participation is essential to address community health problems and, in this context, would be complemented by the involvement of RHMs in the planning of activities for future iterations of the programme. Finally, a reintroduction of the RHM programme by government ministries at the national and regional levels, including community-level implementers (e.g. RHMs, nurses, community leaders), would allow for increased recognition of RHMs, clarification of the role and potentially improve health in rural areas. Addressing these matters represents an important opportunity to recognise and harness the full potential of community-based approaches to public health challenges.

## Limitations

Several limitations of this study have been identified. MSF has a history of training and engaging RHMs, and recruitment of participation was done by MSF staff. Despite efforts, otherwise, most RHMs interviewed had already been trained by MSF on HIV. This may have influenced participants during interviews due to the existing relationships with the organisation. Attempts to mitigate this were made—the objective of the research was emphasised when introducing it, in efforts to de-link MSF and their historical involvement with RHMs. Additionally, only active RHMs were interviewed meaning a fuller perspective of RHMs and their challenges is not possible. At the time of this study, a total of 1 275 RHMs were recorded to be working in the region, of which 15 male RHMs were active but not working in either of the selected study areas. This research therefore only captured the perspective of female RHMs. Finally, in the broader scope, a direct implementation for research focuses on the country of Eswatini and may not be generalisable to all contexts. However, similarities amongst health challenges and CHW experiences in southern Africa have been recognised, meaning this research may serve as a point of reflection for other contexts considering CHW recognition, specifically in rural communities.

## Conclusion

In order to provide recognition for RHMs promoting positive health outcomes in communities, adequate support mechanisms and role clarification is needed. Understanding of community realities is essential to plan successful public health interventions and to recognise community level implementers.

## Data Availability

Due to confidentiality, access to data is restricted to MSF.

## Abbreviations

ACF: Active Case Finder
CHW: Community health worker
FGD: Focus group discussion
IDI: In-depth interview
KI: Key informant
MOH: Ministry of Health
MSF: Médecins Sans Frontières
PI: Principal investigator
RHM: Rural Health Motivator
TB: Tuberculosis

## Footnotes

1 The country is subdivided into rural councils known as Tinkhundla

2 Additional concerns exist that an increased stipend would increase community expectations of RHMs.

## References

[1] Kok MC, Dieleman M, Taegtmeyer M, Broerse JE, Kane SS, Ormel H (2015). Which intervention design factors influence performance of community health workers in low- and middle-income countries? A systematic review. Health Policy Plan.

[2] Geldsetzer P, Vaikath M, De Neve J-W, Bossert TJ, Sibandze S, Bärnighausen T (2017). Household coverage of Swaziland’s national community health worker programme: a cross-sectional population-based study. Trop Med Int Health TM IH.

[3] Geldsetzer P, Vaikath M, De Neve J-W, Bossert TJ, Sibandze S, Mkhwanazi M (2017). Distrusting community health workers with confidential health information: a convergent mixed-methods study in Swaziland. Health Policy Plan.

[4] Geldsetzer P, De Neve J-W, Boudreaux C, Bärnighausen T, Bossert TJ. Improving the performance of community health workers in Swaziland: findings from a qualitative study. Hum Resour Health. 2017 [cited 2019 Feb 15];15. Available from: http://human-resources-health.biomedcentral.com/articles/10.1186/s12960-017-0236-x. 10.1186/s12960-017-0236-x PMC5604406 28923076

[5] Jaskiewicz W, Tulenko K (2012). Increasing community health worker productivity and effectiveness: a review of the influence of the work environment. Hum Resour Health.

[6] World Health Organisation, PEPFAR, UNAIDS. Task shifting - global recommendations and guidelines. Geneva: World Health Organization; 2008.

[7] Singh D, Negin J, Otim M, Orach CG, Cumming R (2015). The effect of payment and incentives on motivation and focus of community health workers: five case studies from low- and middle-income countries. Hum Resour Health.

[8] Geldsetzer P, Vaikath M, De Neve J-W, Bärnighausen T, Bossert TJ (2017). A qualitative and quantitative performance evaluation of Swaziland’s rural health motivator program. F1000Research.

[9] WHO | World Health Statistics 2019: Monitoring health for the SDGs [Internet]. WHO. World Health Organization; [cited 2020 May 12]. Available from: http://www.who.int/gho/publications/world_health_statistics/2019/en/.

[10] Lewin SA, Dick J, Pond P, Zwarenstein M, Aja G, van Wyk B, et al. Lay health workers in primary and community health care. Cochrane Database Syst Rev. 2005;CD004015. 10.1002/14651858.CD004015.pub2 15674924

[11] Bhutta Zulfiqar A, Lassi Zohra S, George Pariyo, Huicho Luis. Global experience of community health workers for delivery of health related millennium development goals: a systematic review, country case studies, and recommendations for integration into national health systems. World Health Organization, Global Health Workforce Alliance.

[12] Ministry of Health (2015). Standard operational guidelines for community based health volunteers in Swaziland.

[13] Mwai GW, Mburu G, Torpey K, Frost P, Ford N, Seeley J (2013). Role and outcomes of community health workers in HIV care in sub-Saharan Africa: a systematic review. J Int AIDS Soc.

[14] Singh P, Sachs JD (2013). 1 million community health workers in sub-Saharan Africa by 2015. Lancet Lond Engl.

[15] De Neve J-W, Garrison-Desany H, Andrews KG, Sharara N, Boudreaux C, Gill R (2017). Harmonization of community health worker programs for HIV: a four-country qualitative study in southern Africa. Tsai AC, editor. PLoS Med.

[16] Lees S, Kielmann K, Cataldo F, Mburu G. Understanding the linkages between informal and formal care for people living with HIV in sub-Saharan Africa. Glob Public Health. 2012;7:1109–19. 10.1080/17441692.2012.733403 23116123

[17] Doherty TM, Coetzee M (2005). Community health workers and professional nurses: defining the roles and understanding the relationships. Public Health Nurs Boston Mass.

[18] Mb M, Bt C, Bh C, A M, Hf C, J M, et al. Use of task-shifting to rapidly scale-up HIV treatment services: experiences from Lusaka, Zambia. BMC Health Serv Res. 2009;9:5–5. 10.1186/1472-6963-9-5 PMC2628658 19134202

[19] Abdel-All M, Abimbola S, Praveen D, Joshi R (2019). What do accredited social health activists need to provide comprehensive care that incorporates non-communicable diseases? Findings from a qualitative study in Andhra Pradesh, India. Hum Resour Health.

[20] UNICEF. Health Budget Brief 2018/2019 Kingdom of Eswatini. Mbabane; 2019.

[21] Monitoring and Evaluation Unit. Community based health services: 2017 annual programme report. Kingdom of Eswatini Ministry of Health;.

[22] World Health Organization. Swaziland: summary country profile for HIV/AIDS treatment scale up. 2005 Dec.

[23] Escott S, Walley J (2005). Listening to those on the frontline: lessons for community-based tuberculosis programmes from a qualitative study in Swaziland. Soc Sci Med 1982.

[24] Green J, Thorogood N (2004). Qualitative methods for health research.

[25] Eswatini Ministry of Health, PEPFAR, CDC, ICAP. Swaziland HIV Incidence Measurement Survey 2: a population-based HIV impact assessment. SHIMS2; 2017.

[26] Palys, T in Given, LM (ed). Purposive sampling. Sage Encycl Qual Res Methods Vol2. Los Angeles: SAGE Publications Ltd; 2008. p. 697–8.

[27] Skovdal M, Cornish F. Qualitative research for development: a guide for practitioners [Internet]. Practical Action Publishing Ltd; 2015 [cited 2019 Feb 24]. Available from: https://www.developmentbookshelf.com/doi/book/10.3362/9781780448534.

[28] Braun V, Clarke V (2006). Using thematic analysis in psychology. Qual Res Psychol.

[29] King N. Using templates in the thematic analysis of text. Essent Guide Qual Methods Organ Res. London: SAGE Publications Ltd; 2004. p. 256–70.

[30] Dlamini-Simelane T (2017). The GIPA concept ‘lost in transition’: the case of expert clients in Swaziland. Anthropol Action.

[31] Najafizada SAM, Bourgeault IL, Labonté R (2019). A gender analysis of a national community health workers program: a case study of Afghanistan. Glob Public Health.

[32] Theobald S, MacPherson EE, Dean L, Jacobson J, Ducker C, Gyapong M, et al. 20 years of gender mainstreaming in health: lessons and reflections for the neglected tropical diseases community. BMJ Glob Health. 2017 [cited 2019 Dec 4];2. Available from: https://gh.bmj.com/content/2/4/e000512. 10.1136/bmjgh-2017-000512 PMC5687534 29177100

[33] Rosie Steege, Kate Hawkins. Gender and community health workers: three focus areas for programme managers and policy makers | CHW Central. [cited 2019 Nov 29]. Available from: https://www.chwcentral.org/gender-and-community-health-workers-three-focus-areas-programme-managers-and-policy-makers.

[34] Buseh AG, Glass LK, McElmurry BJ (2002). Cultural and gender issues related to HIV/AIDS prevention in rural Swaziland: a focus group analysis. Health Care Women Int.

[35] Swaziland Government, The Kingdom of Swaziland. National Gender Policy. 2010.

[36] Musoke D, Ssemugabo C, Ndejjo R, Ekirapa-Kiracho E, George AS (2018). Reflecting strategic and conforming gendered experiences of community health workers using photovoice in rural Wakiso district, Uganda. Hum Resour Health.

[37] Sarin E, Lunsford SS (2017). How female community health workers navigate work challenges and why there are still gaps in their performance: a look at female community health workers in maternal and child health in two Indian districts through a reciprocal determinism framework. Hum Resour Health.

[38] Maes K, Closser S, Tesfaye Y, Abesha R (2019). Psychosocial distress among unpaid community health workers in rural Ethiopia: comparing leaders in Ethiopia’s Women’s development Army to their peers. Soc Sci Med.

[39] Burkot C, Naidi L, Seehofer L, Miles K (2017). Perceptions of incentives offered in a community-based malaria diagnosis and treatment program in the highlands of Papua New Guinea. Soc Sci Med.

[40] Rabbani F, Shipton L, Aftab W, Sangrasi K, Perveen S, Zahidie A (2016). Inspiring health worker motivation with supportive supervision: a survey of lady health supervisor motivating factors in rural Pakistan. BMC Health Serv Res.

[41] Martin SL, Muhomah T, Thuita F, Bingham A, Mukuria AG (2015). What motivates maternal and child nutrition peer educators? Experiences of fathers and grandmothers in western Kenya. Soc Sci Med.

[42] Sommanustweechai A, Putthasri W, Nwe ML, Aung ST, Theint MM, Tangcharoensathien V (2016). Community health worker in hard-to-reach rural areas of Myanmar: filling primary health care service gaps. Hum Resour Health.

[43] George MS, Pant S, Devasenapathy N, Ghosh-Jerath S, Zodpey SP (2017). Motivating and demotivating factors for community health workers: a qualitative study in urban slums of Delhi, India. WHO South-East Asia J Public Health.

[44] Sanou AK, Jegede AS, Nsungwa-Sabiiti J, Siribié M, Ajayi IO, Turinde A (2016). Motivation of community health workers in diagnosing, treating, and referring sick young children in a multicountry study. Clin Infect Dis Off Publ Infect Dis Soc Am.

[45] Oluwole A, Dean L, Lar L, Salami K, Okoko O, Isiyaku S (2019). Optimising the performance of frontline implementers engaged in the NTD programme in Nigeria: lessons for strengthening community health systems for universal health coverage. Hum Resour Health.

